# Prioritizing Chemicals for Risk Assessment Using Chemoinformatics: Examples from the IARC Monographs on Pesticides

**DOI:** 10.1289/EHP186

**Published:** 2016-05-10

**Authors:** Neela Guha, Kathryn Z. Guyton, Dana Loomis, Dinesh Kumar Barupal

**Affiliations:** 1 International Agency for Research on Cancer (IARC) Monographs Programme, and; 2 Section of Nutrition and Metabolism–Biomarkers Group, International Agency for Research on Cancer, Lyon, France

## Abstract

**Background:**

Identifying cancer hazards is the first step towards cancer prevention. The International Agency for Research on Cancer (IARC) Monographs Programme, which has evaluated nearly 1,000 agents for their carcinogenic potential since 1971, typically selects agents for hazard identification on the basis of public nominations, expert advice, published data on carcinogenicity, and public health importance.

**Objectives:**

Here, we present a novel and complementary strategy for identifying agents for hazard evaluation using chemoinformatics, database integration, and automated text mining.

**Discussion:**

To inform selection among a broad range of pesticides nominated for evaluation, we identified and screened nearly 6,000 relevant chemical structures, after which we systematically compiled information on 980 pesticides, creating network maps that allowed cluster visualization by chemical similarity, pesticide class, and publicly available information concerning cancer epidemiology, cancer bioassays, and carcinogenic mechanisms. For the IARC Monograph meetings that took place in March and June 2015, this approach supported high-priority evaluation of glyphosate, malathion, parathion, tetrachlorvinphos, diazinon, p,p′-dichlorodiphenyltrichloroethane (DDT), lindane, and 2,4-dichlorophenoxyacetic acid (2,4-D).

**Conclusions:**

This systematic approach, accounting for chemical similarity and overlaying multiple data sources, can be used by risk assessors as well as by researchers to systematize, inform, and increase efficiency in selecting and prioritizing agents for hazard identification, risk assessment, regulation, or further investigation. This approach could be extended to an array of outcomes and agents, including occupational carcinogens, drugs, and foods.

**Citation:**

Guha N, Guyton KZ, Loomis D, Barupal DK. 2016. Prioritizing chemicals for risk assessment using chemoinformatics: examples from the IARC Monographs on Pesticides. Environ Health Perspect 124:1823–1829; http://dx.doi.org/10.1289/EHP186

## Introduction

The Monographs Programme of the International Agency for Research on Cancer (IARC) has been instrumental in identifying “environmental” factors that can increase the risk of human cancer. Since its inception in 1971, the Monographs Programme has evaluated nearly 1,000 agents (as of 2016) and has classified them with respect to the strength of scientific evidence that they cause cancer in humans.

The IARC Monographs Programme convenes international working groups to identify and classify environmental cancer hazards. The evaluations are based on systematic reviews of epidemiological evidence and cancer bioassay data in experimental animals, with supporting evidence concerning the carcinogenic mechanisms that may act in humans. The sources and extent of human exposure, as well as existing regulations, are also reviewed (IARC 2015). Agents are selected for evaluation by the IARC Monographs Programme through a process that has traditionally relied, to a large extent, on expert recommendations. A public call for nominations of agents is posted on the IARC website, and additional nominations are solicited from participating states, the scientific community (including IARC staff), and the general public. An advisory group is assembled every 5 years to review the nominated agents (Straif et al. 2014) and to assign them low, medium, or high priority for eventual evaluation. These priority levels reflect the committee’s rankings based on the availability of new data, evidence of carcinogenicity, extent of human exposure, and public health importance (IARC 2015). This method has proved useful for identifying agents of public health importance as priorities for the evaluation of their carcinogenic potential. Following this advice, the Programme selects, groups, and orders the priority agents into a series of Monographs, based on a systematic and objective review of the available evidence. Considerations include the priority level assigned by the IARC advisory group, whether and when a compound was last reviewed by IARC and the potential for the classification to change, usage data (including regulations on use) and the extent of human exposure worldwide, the compound’s classification by other agencies, and the volume and complexity of informative data that can reasonably be considered during the course of an IARC Monograph meeting. Another important consideration is public health concern, including possible impacts in low- and middle-income countries.

The most recent IARC Advisory Group recommended that the Monographs Programme evaluate pesticides. Nominations included specific compounds, chemical classes, and related occupations. Some pesticides have been identified as potential human carcinogens by authoritative bodies, including IARC, but many are either lacking evaluations, or the evaluations may be outdated. Specifically, compounds of the organophosphate, organochlorine, triazine, carbamate, dinitroaniline, and pyrethroid classes were accorded priority in 2014 (Straif et al. 2014).

Pesticides include thousands of unique chemical structures distributed across broad chemical and functional classes. Many are chemically or functionally related, but the extent to which they have been studied and the amount of information available from public databases (e.g., PubMed, the Tox21 Program, the PubChem bioactivity assay database) differs markedly across compounds. Given the large amount of data and the structural diversity between compounds, manual review may be prone to incomplete coverage, bias, and low efficiency.

Automation of literature mining, integration of electronically available databases, and advanced data visualization could be employed as a complementary approach to systematically incorporate chemical similarity as well as to identify the extent of available information. To address the challenge of appropriately grouping agents and ordering recommended priorities for hazard assessment, we present a systematic and objective approach using chemoinformatics that has been used to select pesticides for evaluation in recent IARC monographs (Guyton et al. 2015; Loomis et al. 2015).

## Methods

### Overview

A bioinformatics approach was used to systematically assemble and visualize the extent of available information according to chemical similarity across pesticide active compounds. The ranking obtained from the bioinformatics approach was later compiled manually (see Tables 1 and 2) with other important factors considered in selecting priorities (see the considerations listed in “Introduction”) as shown in Table 1, particularly the assigned priority and the availability of new data to update a previous IARC evaluation.

**Table 1:** High-ranking organophosphate, organochlorine, and chlorophenoxy pesticides identified using a chemoinformatics approach.

Name	Rank	PubMed cancer hits	PubMed human cancer hits	IARC advisory group priority	Notes, including other classifications	Usage notes	Prior IARC classification (year)	2015 IARC classification
Organophosphates
Parathion	1	42	6	—	U.S. EPA Group C (1991)^*a*^	Restricted^*b*^	3 (1987)	2B
Malathion	2	40	12	High	U.S. EPA Suggestive (2000)^*a*^	High^*c*^	3 (1987)	2A
Chlorpyrifos	3	38	14	Medium	U.S. EPA Group E (1993)^*a*^	High^*c,d*^	3 (1987)
Dichlorvos	4	35	12	—	U.S. EPA Suggestive (2000)^*a*^	Some current uses^*e*^	2B (1991)
Diazinon	5	30	16	High	U.S. EPA Not likely (1997)^*a*^	High^*c*^	None	2A
Glyphosate	7	21	9	Medium	U.S. EPA Group C (1985), Group E (1991)^*a*^	High^*d*^^,^^*f*^	None	2A
Tetrachlorvinphos	13	6	1	—	U.S. EPA Likely (2002)^*a*^	Currently used	3 (1987)	2B
Organochlorines
DDT	1	494	190	Medium	POP^*g*^, RoC-RA^*h*^	Restricted^*b*^	2B**(1991)	2A
Lindane	2	189	51	High	POP^*g*^, RoC-RA^*h*^; U.S. EPA Suggestive (2001)^*a*^	Restricted^*b*^	2B (1987)	1
Dieldrin	3	151	57	—	POP^*g*^	Restricted^*b*^	3 (1987)
Aldrin	7	56	25	—	POP^*g*^	Restricted^*b*^	3 (1987)
Chlorophenoxy
2,4-Dichlorophenoxy acetic acid	1	145	84	—	U.S. EPA Group D (2004)^*a*^	High^*d*^^,^^*e*^	None	2B
Abbreviations: DDT, *p*,*p*′-dichlorodiphenyltrichloroethane; POP, persistent organic pollutant; RoC-RA, reasonably expected to be a human carcinogen. ^***a***^For a description of U.S. EPA cancer classifications, see https://www.epa.gov/fera/risk-assessment-carcinogens. ^***b***^Severely banned or restricted for health or environmental reasons (Rotterdam Convention, Annex III; http://www.pic.int/theconvention/chemicals/annexiiichemicals/tabid/1132/language/en-us/default.aspx). ^***c***^Among the most commonly used OP insecticides in all U.S. market sectors 2001–2007 (https://www.epa.gov/sites/production/files/2015-10/documents/market_estimates2007.pdf). ^***d***^Among the most commonly used conventional pesticide active ingredients in the United States (https://www.epa.gov/sites/production/files/2015-10/documents/market_estimates2007.pdf). ^***e***^Many domestic and other uses of dichlorvos in the United States have been discontinued (U.S. EPA 1995). ^***f***^Most commonly used conventional pesticides in the agricultural sector (glyphosate) or non-agricultural (2,4-D) market sectors (https://www.epa.gov/sites/production/files/2015-10/documents/market_estimates2007.pdf). ^***g***^Listed as a POP under the Stockholm Convention (http://chm.pops.int/). ^***h***^Listed as “reasonably expected to be a human carcinogen” in the U.S. Report on Carcinogens (http://ntp.niehs.nih.gov/pubhealth/roc/roc13/index.html).								

**Table 2:** Cancer epidemiology literature retrieved through automated text mining (chemoinformatics) and manual PubMed searches for pesticides evaluated in IARC Monographs 112 and 113.

Pesticide	Chemoinformatics	Manual searches		
	Retrieved	Retrieved	Included	Excluded
Malathion	12	80	28	52
Parathion	6	12	9	3
Diazinon	16	39	22	17
Tetrachlorvinphos	1	4	4	0
Glyphosate	9	50	19	31
DDT	190	224	116	107
Lindane	51	46	22	24
2,4-D	84	76	62	11
Abbreviations: 2,4-D, 2,4-dichlorophenoxyacetic acid; DDT, *p*,*p*′-dichlorodiphenyltrichloroethane.				

**Figure 1:**
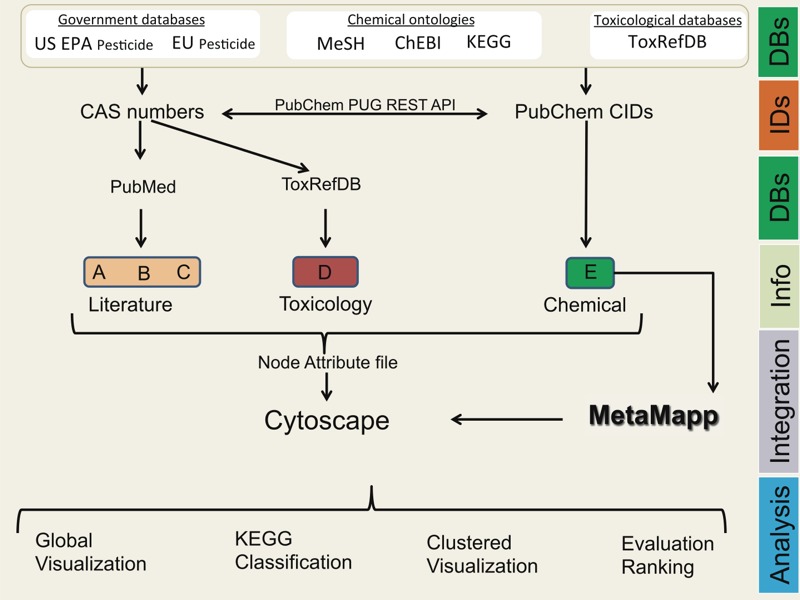
Overall scheme of the chemoinformatics approach for data retrieval and visualization for the prioritization of pesticides for the evaluation of their carcinogenic potential. A, PubMed Cancer All; B, PubMed Cancer Epidemiology; C, PubMed animal cancer bioassays; D, ToxRefDB carcinogenicity; E, Chemical Similarity Scores. See Table S2 for a description of the search terms. Notes: CAS, Chemical Abstracts Service; ChEBI, Chemical Entities of Biological Interest; CID, Compound Identifier; EU, European Union; KEGG, Kyoto Encyclopedia of Genes and Genomes; MeSH, Medical Subject Headings; PUG REST API, power user gateway representational state transfer application programming interface; ToxRefDB, Toxicity Reference Database.

The first step was to compile a list of all pesticide compounds, which was then organized into chemical similarity network maps. To visualize the availability of data on all pesticides, information by topic area that is considered for an IARC Monograph evaluation (cancer epidemiology, cancer bioassays in animals, mechanistic studies) was then overlaid onto the network maps. Chemical network maps were generated by integrating lists of pesticide compounds with their chemical structures and subsequently mining public databases of the published literature. This process is documented in Figure 1 and is detailed in the following sections.

### Creation of a Master List of Pesticides

We compiled a master list of pesticide compounds—including chemical name, chemical class, and Chemical Abstract Service (CAS) number—from the Kyoto Encyclopedia of Genes and Genomes (KEGG), Medical Subject Heading (MeSH) Pesticides, European Union (EU) pesticides, Chemical Entities of Biological Interest (ChEBI) pesticides, U.S. Environmental Protection Agency (EPA) Pesticides, and the U.S. EPA Toxicity Reference (ToxRef) databases. Chemical structures for each pesticide compound were obtained by linking CAS numbers to PubChem Compound Identifiers (CID) (https://pubchem.ncbi.nlm.nih.gov/search/help_search.html) (see Table S1).

### Data Retrieval from National Center for Biotechnology Information Databases

To assess the scope of the published literature for each pesticide, we searched the titles and abstracts of publications catalogued in PubMed on cancer epidemiology and animal bioassays using the CID, the CAS number, and various search terms (see Table S2). The MeSH term “neoplasm” was used for these searches because the keyword “cancer” frequently retrieves false positive hits. The papers manually retrieved in our study were also retrieved using the “neoplasms[MeSH]” query, indicating that the query covers relevant papers. Searches of the ToxRef database (ToxRefDB) and of PubChem bioassays were also conducted (see Table S2). National Center for Biotechnology Information (NCBI) E-utilities and PubChem power user gateway representational state transfer (PUG REST) web services were used to systematically query the databases to obtain results of literature and bioassays searches (Figure 1). Automation for retrieval of data from APIs (application programming interface) was achieved in NodeJS software (https://nodejs.org/en/) using JavaScript programming language. To rank pesticides using the chemoinformatics approach, the pesticides were sorted by chemical class, the number of publications on cancer and that pesticide overall, the number of cancer epidemiology publications, and the information in ToxRefDB (present or absent).

### Chemical Similarity Network Visualization

Network graphs for the chemicals were created using MetaMapp software (Barupal et al. 2012) and visualized in Cytoscape software version 3.1 [U.S. National Institute of General Medical Sciences (http://www.cytoscape.org/release_notes_3_1_1.html)]. Individual pesticides are represented as nodes on the chemical similarity maps. Two nodes were linked in the chemical network graph if their Tanimoto similarity score (a coefficient of similarity between two molecules, a measure commonly used in chemoinformatics) was > 0.60, indicating > 60% chemical similarity. The length of the line connecting the nodes had no meaning itself; it was drawn in reference to the nodes it connected. The node positions within the network maps were controlled by the organic layout algorithm in Cytoscape, which considered a node’s degree (the number of connections to a node) and its clustering coefficient (the ratio of the number of actual connections to the total number of possible connections among the node and its neighbors).

A global network of all the pesticides and two focused network graphs of the organophosphorus (OP) and organochlorine (OC) pesticide classes were created. Beyond the KEGG classification, we broadened the pesticide categories for visualization by including pesticides with at least one phosphorous atom or two chlorine atoms to the OP and OC pesticide classes, respectively. These network graphs and the data table used to generate the graphs are provided online at http://pesticide.barupal.org/.

### Automated Text Mining Versus Directed Literature Searches

For the top-ranking chemicals identified via the chemoinformatics approach, we compared the results from the automated searches to directed PubMed searches. The comparison focused on cancer epidemiology because most such studies are found in the published literature. The comparison also considered any published animal cancer bioassays and studies of key mechanistic evidence (Smith et al. 2016) relating to the carcinogenicity of the compound. The manual literature searches and screening were performed using The Health Assessment Workspace Collaborative (Shapiro 2015).

## Results

### Creation of a Master Pesticide List for Literature Mining

A master list of nearly 6,000 pesticide compounds was created from governmental databases, ontologies, and databases providing toxicological data on chemicals: KEGG (*n* = 916 pesticides), MeSH Pesticides (*n* = 451 pesticides), ChEBI (*n* = 2,448 pesticides), U.S. EPA Pesticides (*n* = 5,774 pesticides), EU Pesticides (*n* = 1,318 pesticides) and ToxRefDB (*n* = 474). Entries that were imported from these databases were excluded from the final list if *a*) the structures represented additives such as ethanol; *b*) they did not have a CAS Registry Number (CASRN) or a PubChem CID; *c*) they were not present in at least three of the aforementioned databases; or *d*) they were compounds with applications in multiple industries, such as phenol, nicotine, acrolein, and bisphenol A. All of the compounds from the KEGG, ToxRef, and MESH Pesticide databases were included in the analysis, but a number of entries were excluded from the U.S. EPA (*n* = 5,024), ChEBI (*n* = 2,033), and EU Pesticides databases (*n* = 643). KEGG provided chemical classification information, and ToxRefDB provided toxicological data, in particular cancer bioassay data, for approximately 400 selected pesticides. The final list contained 980 pesticide structures (see http://pesticide.barupal.org/ and http://pesticide.barupal.org/dataTable.html).

### Selection of Pesticides for Evaluation in IARC Monograph Volumes 112, 113, and 117

**Figure 2:**
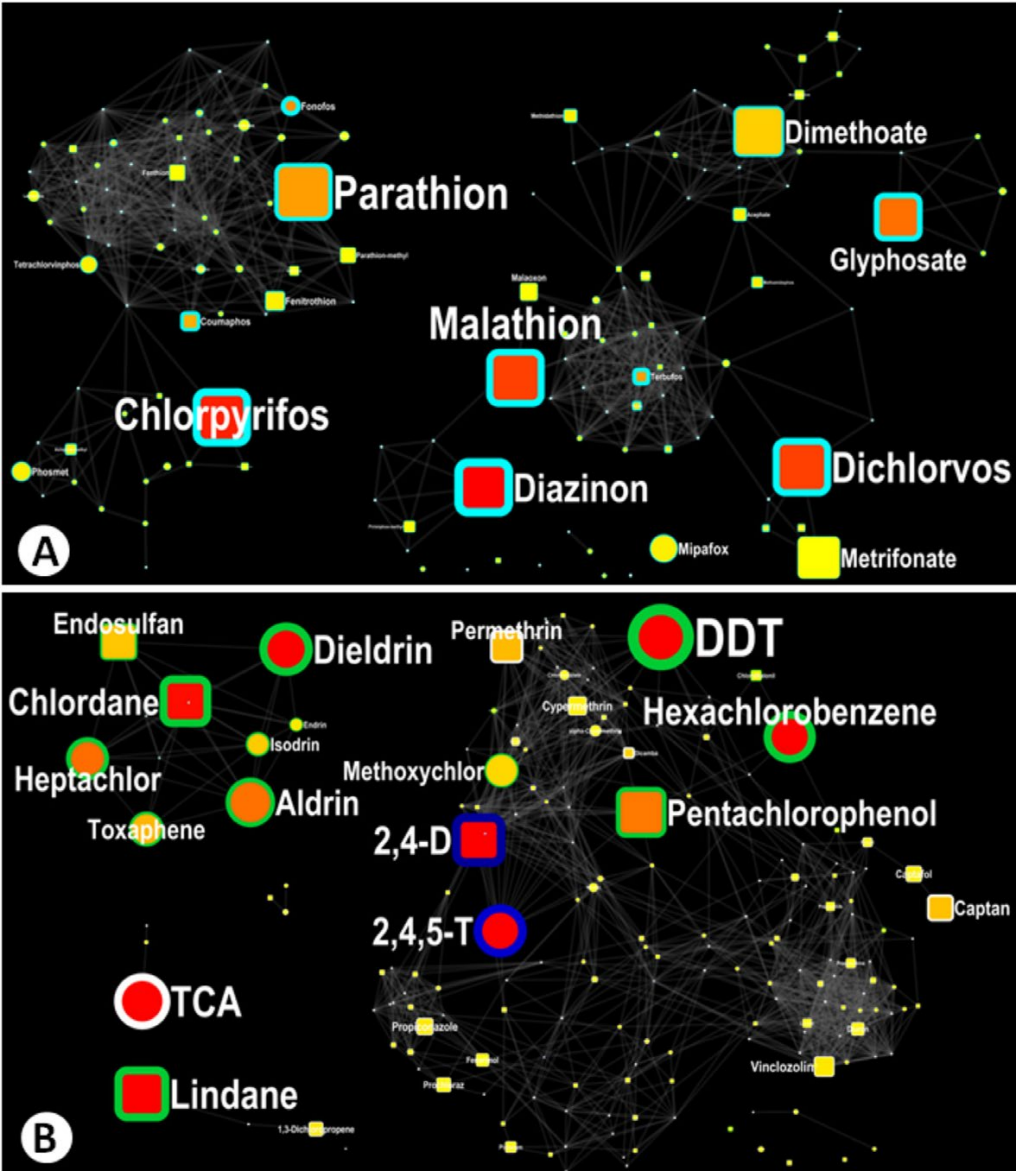
Focused visualization of the chemical similarity network maps for (*A*) organophosphorus and other pesticides with at least 1 phosphorous atom and (*B*) organochlorines and other pesticides with more than 2 chlorine atoms. Individual pesticides are represented as nodes on the chemical similarity maps. The node size is proportional to the number of publications overall on a pesticide and cancer: larger nodes represent more publications. The node border width represents the number of publications on epidemiology, cancer, and the pesticide: a thicker border represents more papers. The node color also represents the number of publications on epidemiology, cancer, and the pesticide: red represents the highest count of publications. The node shape indicates whether results for a particular pesticide were available in the Toxicity Reference Database (ToxRefDB) (circle = absent; square = present). The node border color represents the Kyoto Encyclopedia of Genes and Genomes (KEGG) pesticide classification. Greater detail on the colors used as well as the associated table describing the information in the figures can be found online (http://pesticide.barupal.org/). Two nodes are linked by a line if their Tanimoto similarity score is > 0.60 (hence, they are > 60% chemically similar). The length of the line connecting the nodes has no meaning itself; it is drawn in reference to the nodes it is connecting. The node positions within the network maps are controlled by the organic layout algorithm in Cytoscape software (http://www.cytoscape.org/release_notes_3_1_1.html), which considers a node’s degree (the number of connections to a node) and its clustering coefficient (the ratio of the number of actual connections to the total number of possible connections among the node and its neighbors). The session file that can be opened in Cytoscape for zoom-in and focused visualization is located online (http://pesticide.barupal.org/).

The preceding approach was a starting point for selecting pesticides for evaluation in IARC Monographs volumes 112, 113, and 117 (described below). Using this approach, many of the top-ranked pesticides belonged to the organophosphate (OP) and organochlorine (OC) classes; therefore, these pesticide classes were accorded priority. To rank pesticides using the chemoinformatics approach, the pesticides were sorted by chemical class, the overall number of publications on cancer and that pesticide, the number of cancer epidemiology publications, and the information in ToxRefDB (present or absent) (Table 1). The chemical network maps of OPs (Figure 2A) and OCs (Figure 2B) were informative about chemical similarities across potential candidates and were useful for identifying related compounds that might be evaluated as a mechanistic class.

In addition to the ranking of OPs and OCs obtained using the chemoinformatics approach (Table 1), several other criteria were considered in order to select a subset of pesticides for evaluation in Monographs 112 and 113 (see the considerations listed in “Introduction”) as indicated in Table 1, particularly the assigned priority and the availability of new data to update a previous IARC evaluation. The volume and complexity of informative data are important determinants of the number and diversity of agents that can be evaluated in a Monograph meeting. The chemoinformatics approach was therefore useful for visualizing the volume of literature by topic area (cancer epidemiology, animal cancer bioassays, supporting mechanistic evidence). To further refine the list of agents for evaluation, additional directed PubMed searches for epidemiologic and mechanistic data were conducted using standard search strings developed for the IARC Monographs.

### Organophosphate Pesticides

Based on the preceding criteria, parathion, malathion, diazinon, glyphosate, and tetrachlorvinphos emerged as promising candidates for new evaluation in IARC Monograph Volume 112 (Figure 2B). Parathion and malathion, the top-ranked OPs identified by the chemoinformatics approach, were previously evaluated by the IARC Monographs in 1987 and were then assigned to Group 3 (not classifiable). However, they were later (in 1991 and 2000, respectively) classified by the U.S. EPA as potential carcinogens on the basis of positive bioassay data. The availability of newly published epidemiologic studies, particularly for malathion, also supported their selection for re-evaluation in Volume 112. Diazinon, like malathion, was assigned high priority for evaluation by an international advisory group to the IARC Monograph Progamme. Diazinon was ranked fifth by the chemoinformatics approach, had the most cancer epidemiology studies among the OPs, and had not been previously evaluated by the IARC Monographs Programme.

In contrast, several candidate agents did not appear to have animal bioassay or other evidence to support a different (e.g., dichlorvos, Group 2B; chlorpyrifos, Group 3) or new (e.g., dimethoate) IARC evaluation. These included several compounds classified by the U.S. EPA in “Group E–Evidence of non-carcinogenicity” (terbufos, fonofos, chlorpyrifos and phorate), indicating that animal bioassays had been performed, but none had positive findings (U.S. EPA 2015). An additional factor, noted in Advisory Group recommendations, is that although new epidemiological evidence has emerged, it remains incomplete for these agents. It will be important to obtain the results from ongoing analyses (e.g., those being conducted by the Agricultural Health Study or the AGRICOH consortium), before performing any new or updated evaluations. Thus, these candidates were accorded a lower ranking overall for near-term evaluation.

In contrast, a recent meta-analysis identified relevant epidemiologic findings for glyphosate (Schinasi and Leon 2014). Additionally, a 1985 classification by the U.S. EPA in Group C for glyphosates indicated the possible availability of pertinent bioassay data (http://www.epa.gov/iris). Glyphosate, ranked seventh by the chemoinformatics approach, was assigned medium priority for near-term evaluation by the Advisory Group. The high production volume of glyphosate leads all OPs and all herbicides, and exposures are widespread, which was another factor in its inclusion among the agents in Volume 112. Although it has some structural similarity to other OPs, glyphosate is toxicologically dissimilar and lacks cholinesterase-inhibiting activity.

Another compelling candidate that emerged from the chemoinformatics approach was tetrachlorvinphos (ranked 13). Tetrachlorvinphos is currently in use, although its overall production volume is low. Tetrachlorvinphos was previously evaluated by the IARC Monographs in 1987 and was then assigned to Group 3 (not classifiable); however, it was later (in 2002) classified by the U.S. EPA as a likely human carcinogen based on positive cancer bioassays. Additionally, because tetrachlorvinphos is a direct-acting oxon, *in vitro* tests for bioactivity might be more informative for tetrachlorvinphos than for other compounds (e.g., malathion) that require metabolic activation to their oxon forms. These mechanistic considerations, together with the positive cancer bioassay findings, were the basis for its inclusion in Volume 112. This selection also took into account the overall volume of literature available on the other four compounds, with the relatively small amount of tetrachlorvinphos literature overall making it feasible to include.

For the five selected compounds, IARC queried governments and requested the public release of government reports on animal cancer bioassays and other relevant data (e.g., genotoxicity) that had been developed by industry. Direct literature searches identified recently reported epidemiological data, including case–control and cohort studies in the United States, Canada, Europe, and Sweden. Directed literature searches also identified studies examining relevant carcinogenic mechanisms, including genotoxicity, for both the parent compounds (e.g., malathion, diazinon) and their oxon metabolites. Recent high-throughput data also provided new insights into the extent of biological activity.

In all, these considerations supported the selection of compounds accorded high (malathion, diazinon) or medium (glyphosate) priority for evaluation by the Advisory Group, as well as two others (parathion and tetrachlorvinphos) that were not specifically highlighted in the broad recommendation to evaluate pesticides.

### Organochlorine Pesticides and 2,4-D

Among the OC pesticides, *p*,*p*′-dichlorodiphenyltrichloroethane (DDT), lindane, aldrin, and dieldrin were identified as promising candidates for new evaluation according to the criteria described above (Figure 2B). DDT was particularly notable as the pesticide with the largest number of human cancer studies and the largest overall number of PubMed articles retrieved (see table online at http://pesticide.barupal.org/dataTable.html). DDT was previously evaluated by IARC in 1991 and had a large number of new studies published in the intervening years; lindane had only been evaluated as part of the broader class of hexachlorocyclohexanes and also had new data; and aldrin and dieldrin were last evaluated in 1987, but few human studies were available (Table 1). All four are listed as persistent organic pollutants under the Stockholm Convention. DDT and lindane were assigned medium and high priority, respectively, for evaluation by the IARC advisory group, and both had previously been listed as “reasonably anticipated to be a human carcinogen” in the National Toxicology Program (NTP) Report on Carcinogens (NTP 2014). No additional classifications were identified for aldrin or dieldrin.

A notable literature database was also available for the herbicides 2,4-D and 2,4,5-trichlorophenoxyacetic acid (2,4,5-T) (Figure 2B), which, in 1987, were classified by IARC in Group 2B as part of the class of chlorophenoxy herbicides (IARC 1987). However, because 2,4,5-T is frequently contaminated with dioxin, which is already classified in IARC Group 1 (IARC 2012), it was not considered further.

As was done for the OP pesticides, directed literature searches identified relevant epidemiologic and mechanistic studies and cancer bioassays for the OC pesticides. Additionally, we requested public release of information on cancer bioassays conducted with these compounds that were not available in the public domain.

After considering feasibility, including the unusually large volume of data retrieved for DDT, in addition to the preceding scientific issues, lower-ranked aldrin and dieldrin were set aside from the list of potential candidates for later evaluation in IARC Monograph Volume 117 (October 2016), whereas DDT and lindane were selected for evaluation in Volume 113. In addition, 2,4-D was selected after considering both its widespread use and the volume of published literature available (Table 1).

### Directed Literature Searches for Pesticides Prioritized for Evaluation

For the pesticides prioritized for evaluation by IARC in Monograph Volumes 112 and 113 based on the chemoinformatics approach, manual searching and screening of the epidemiological literature was performed using the Health Assessment Workspace Collaborative (https://HAWCproject.org). Such manual validation is supported because the amount of the published literature—particularly concerning epidemiology and carcinogenic mechanisms—does not always predict the need for a new or updated evaluation. On the other extreme, even a single new well-conducted cancer bioassay could justify further evaluation. Accordingly, the findings from the manual screening were compared with the results from the automated searches to determine the relevance of retrieved articles to any resulting evaluation (Table 2; see also Table S3).

In general, the chemoinformatics approach retrieved fewer cancer epidemiology papers than were identified through manual searches. For example, for DDT, 190 cancer epidemiology papers were identified through automated searches in PubMed. In comparison, 224 were identified through targeted manual searches, of which 116 were included after manual review as relevant to an evaluation. Automated searches were not performed on the literature on cancer mechanisms because methods to comprehensively identify the broad range of cancer-relevant mechanistic data for potential carcinogens have only recently been advanced (Smith et al. 2016). Nevertheless, targeted searches developed according to the principles outlined by Smith et al. (2016) identified a substantial volume of articles on each selected compound. For instance, targeted manual searches and screening of the mechanistic literature for the evaluation of organophosphate pesticides identified relevant publications on malathion (*n* = 370), parathion (*n* = 578), diazinon (*n* = 215), tetrachlorvinphos (*n* = 40) and glyphosate (*n* = 204). Yet more articles were included for the subsequent evaluation of DDT (*n* = 953), lindane (*n* = 545), and 2,4-D (*n* = 420). Overall, this exercise demonstrated that the chemoinformatics approach not only provided an efficient and accurate indication of the size of the relevant literature but also identified studies that would be relevant to any resulting evaluation.

## Discussion

We present a novel method to select agents for hazard identification that has been applied in the IARC Monographs Programme. Although the data used to construct the chemical network graphs are publicly available, they had not previously been organized in a unified manner that would allow for the simultaneous analysis of the volume of literature on a particular chemical or group of related chemicals. Beyond the KEGG classification, we broadened the pesticide categories for visualization by including pesticides with at least one phosphorous atom or two chlorine atoms in the OP and OC pesticide classes, respectively. Doing so enabled us to map pesticides by chemical similarity and to include pesticides that may have been missed by pesticide databases or were discarded owing to an error in assignment of chemical class. Accordingly, using a chemoinformatics approach, we were able to integrate information on chemical structure similarity for 980 compounds with the results of systematic, automated text mining of cancer-relevant published information in public databases. The use of web technologies streamlined the integration of information retrieved from different databases/sources and improved efficiency through creating network maps to visualize both key chemicals and chemicals that were less studied but chemically related and that may act through a similar mechanism. By enhancing the visualization of large-scale public data, our chemoinformatics approach can complement other technologies that employ biomedical text–mining strategies to support cancer risk assessment and research (Korhonen et al. 2012).

Using this chemoinformatics approach, pesticides in the organophosphate and organochlorine classes were accorded priority for evaluation in IARC Monograph Volumes 112 and 113: malathion, parathion, diazinon, glyphosate, tetrachlorvinphos (Guyton et al. 2015), DDT, lindane, and 2,4-D (Loomis et al. 2015). In the resulting evaluations, all of these pesticides were assigned a new or higher IARC classification, reflecting the adequacy of the identified evidence to support these cancer hazard evaluations. In particular, three pesticides previously assigned to Group 3 (not classifiable) were classified in Group 2B (parathion, tetrachlorvinphos) or Group 2A (malathion). Likewise, two others were reclassified from Group 2B to Group 2A (DDT) or Group 1 (lindane). 2,4-D was newly classified in Group 2B; previously, IARC had classified the entire class of chlorophenoxy herbicides as Group 2B. Finally, two pesticides, diazinon and glyphosate, both assigned to Group 2A, had not been previously classified by IARC. For several of these compounds (including malathion, diazinon, glyphosate, DDT, lindane, and 2,4-D), strong mechanistic evidence supported the resulting evaluations. In all, these results affirm the utility of the prioritization method for identifying compounds that have evidence warranting new or updated IARC Monograph evaluations.

In addition to the pesticides selected for evaluation in IARC Monograph Volumes 112 and 113, the chemoinformatics approach highlighted several other compounds or compound classes (see http://pesticide.barupal.org/). Several of these have been previously assigned by the IARC Monographs Programme (IARC 2016) to Group 2B or higher including trichloroacetic acid (Group 2B, 2014), inorganic arsenic compounds (Group 1, 2012), hexachlorobenzene (Group 2B, 2001), and polychlorphenols (Group 2B, 1999). Others previously assigned to Group 3 and of high use, including atrazine (Group 3, 1999), could be immediately prioritized for re-evaluation together with related compounds (i.e., simizine). Indeed, atrazine was accorded medium priority by the expert advisory group (Straif et al. 2014) based on extensive use and exposures, as well as on suspicion of carcinogenicity suggested by newly published information; furthermore, the chemoinformatics method also indicated an extensive literature base. Similarly, diverse compounds currently assigned to IARC Group 3 also emerged (e.g., captan, 1987; methyl bromide, 1999; piperonyl butoxide, 1987), evidently based on information published since the last IARC evaluation. These compounds include some of the most frequently used conventional pesticide active ingredients (e.g., the fumigant methyl bromide, ranked as the 8th (in 2007, 2005 and 2003) or 7th (in 2001) most commonly used conventional pesticide active ingredient by the U.S. EPA (Grube et al. 2011). As noted above for the agents selected for evaluation in Volume 112, chlorpyrifos and other compounds of the organophosphate class may merit re-evaluation following completion and publication of important epidemiological evaluations. Interestingly, some compounds that emerged as having relevant studies for cancer hazard evaluation have not been previously evaluated or specifically nominated for IARC evaluation (e.g., paraquat).

In general, the chemoinformatics approach retrieved fewer cancer epidemiology papers than were identified through the directed literature searches. There are several possible explanations for this discrepancy. Some articles may have been missed because automated searches were of the article title and abstract, whereas epidemiology papers sometimes report on multiple pesticides, and specific compounds may not have been listed in the title or abstract. The automated searches relied on MeSH annotation using “neoplasms[mesh]” as a more precise search term instead of the keyword “cancer.” This keyword could potentially retrieve irrelevant papers (e.g., papers that do not describe a laboratory or epidemiological finding on cancer) that MeSH terms would filter. Nonetheless, there may be delays in assigning publications MeSH annotations; thus, more recent but still relevant papers may not be retrieved. The directed searches scanned the full text of publications, enabling identification of publications not retrieved by automated searches using only MeSH terms. A potential limitation of the automated text-mining approach is that the search is more specific but less sensitive, sometimes necessitating a manual validation of the literature base to ensure that all relevant publications have been captured. However, this limitation primarily affects the later stages of literature retrieval rather than the initial planning phase. For future efforts using the chemoinformatics approach, we could increase the sensitivity of the automated search by broadening the search terms to include key terms identified from the most informative studies found in a manual search.

Demand for the evaluation of potential chemical hazards is increasing at the present time, while the resources for testing these chemicals are decreasing (Benigni et al. 2013). The use of long-term cancer bioassays in animals, which have previously played a fundamental role in chemical hazard assessment, is declining for ethical and practical reasons (e.g., concern for animal welfare, expense, time). Therefore, alternative, cost-effective strategies for predicting the toxicological properties of chemicals, such as (Quantitative) Structure–Activity Relationships [(Q)SAR], are being proposed and supported by regulatory initiatives such as Registration, Evaluation, Authorisation and Restriction of Chemicals (REACH) (http://ec.europa.eu/environment/chemicals/reach/reach_en.htm) (Benigni et al. 2013; van Leeuwen et al. 2009). These approaches capitalize on the wealth of data already captured in publicly available databases.

By employing technological advances in bioinformatics and computational toxicology, we have demonstrated that the use of chemoinformatics is a powerful and complementary approach for prioritizing chemicals for risk assessment. This approach could be further extended to support prediction of emerging risks and informed substitution of hazardous chemicals with “safer” alternatives (Jacobs et al. 2016), wherein bioinformatics approaches could be used to compare compounds that have not yet been tested, but are structurally similar, to agents already classified for their carcinogenic potential by the IARC Monographs Programme. Epidemiologists and other researchers assessing associations between numerous chemicals and outcomes may also be able to employ this strategy to identify agents for further investigation when designing large-scale studies of human health. Because national health agencies use information from the IARC Monographs as scientific support for their actions to prevent or reduce exposure to potential carcinogens, efficiently prioritizing agents for risk assessment and predicting emerging hazards are important steps towards protecting public health.

## Conclusion

Using a novel chemoinformatics approach, we integrated information on chemical structure similarity with the results of systematic, automated text mining of cancer-relevant information in public databases to select chemical agents for hazard identification. We demonstrate this as an efficient method for grouping of chemicals within class in selecting agents for hazard evaluation in the IARC Monographs. This systematic approach, accounting for chemical similarity and overlaying multiple data sources, can be used to systematize, inform and increase efficiency in selecting and prioritizing agents for hazard identification, risk assessment and regulation or further investigation. Further, by overlaying new chemicals onto a network map of agents that have already been classified by the IARC Monographs, emerging risks and potential cancer hazards (e.g., occupational carcinogens, drugs, environmental pollutants, nutritional compounds) might be identified. This innovation could be extended to an array of outcomes and agents and may prove particularly useful to national regulatory agencies for prioritizing agents for risk assessment and regulation.

## Supplemental Material

(127 KB) PDF
