# Supplementary material for: Prioritizing Chemicals for Risk Assessment Using Chemoinformatics: Examples from the IARC Monographs on Pesticides
Source: Environ Health Perspect. 2016 May 10;124(12):1823–9. doi: 10.1289/EHP186 (PMC5132635; doi:10.1289/EHP186)
Supplement: (127 KB) PDF [file EHP186.s001.acco.pdf]

Environ Health Perspect
DOI: 10.1289/EHP186

Note to readers with disabilities: *EHP* strives to ensure that all journal content is accessible to all readers. However, some figures and Supplemental Material published in *EHP* articles may not conform to 508 standards due to the complexity of the information being presented. If you need assistance accessing journal content, please contact ehp508@niehs.nih.gov. Our staff will work with you to assess and meet your accessibility needs within 3 working days.

Supplemental Material

Prioritizing Chemicals for Risk Assessment Using Chemoinformatics: Examples from the IARC Monographs on Pesticides

Neela Guha, Kathryn Z. Guyton, Dana Loomis, and Dinesh Kumar Barupal

Table of Contents

Table S1. Details of databases on pesticides that were used for the analysis.

Table S2. Search terms used to query databases to assess the scope of the published literature on pesticides.

Table S3. Cancer epidemiology papers retrieved from directed literature searches for parathion.

Environ Health Perspect
DOI: 10.1289/EHP186

Table S1. Details of databases on pesticides that were used for the analysis.

DB Name	Type	Counts of Entries	Web-address
KEGG	Ontology	916	https://www.ncbi.nlm.nih.gov/pccompound?DbFrom=pchierarchy&Cmd=Link&Db=pccompound&LinkName=pchierarchy_pccompound&IdsFromResult=63471
ChEBI	Ontology	2448	https://www.ncbi.nlm.nih.gov/pccompound?DbFrom=pchierarchy&Cmd=Link&Db=pccompound&LinkName=pchierarchy_pccompound&IdsFromResult=984650
Mesh	Ontology	451	https://www.ncbi.nlm.nih.gov/pccompound?DbFrom=pchierarchy&Cmd=Link&Db=pccompound&LinkName=pchierarchy_pccompound&IdsFromResult=1450118
USEPA	Substance Registry	5774	http://iaspub.epa.gov/apex/pesticides/f?p=CHEMICALSEARCH:1:0::NO:1::
EU Pesticide Database	Substance Registry	1318	http://ec.europa.eu/food/plant/pesticides/eu-pesticides-database/public/?event=activesubstance.selection&language=EN
ToxRefDB	Toxicology Database	474	http://www.epa.gov/ncct/toxrefdb/

DOI: 10.1289/EHP186

Table S2. Search terms used to query databases to assess the scope of the published literature on pesticides.

Search	Database API	Query pattern using JavaScript
Cancer All	NCBI Eutils	http://eutils.ncbi.nlm.nih.gov/entrez/eutils/esearch.fcgi?db=pubmed&retmode=json&term=neoplasms[MeSH Terms] AND (*Mesh Descriptor of Pesticide*[MeSH Terms] OR "Chemical Name of Pesticide"[Title/Abstract])
Cancer Animal Studies	NCBI Eutils	http://eutils.ncbi.nlm.nih.gov/entrez/eutils/esearch.fcgi?db=pubmed&retmode=json&term=neoplasms[MeSH Terms] AND animals[Mesh Terms:noexp] AND (*Mesh Descriptor of Pesticide*[MeSH Terms] OR "Chemical Name of Pesticide"[Title/Abstract])
Cancer Epidemiology	NCBI Eutils	http://eutils.ncbi.nlm.nih.gov/entrez/eutils/esearch.fcgi?db=pubmed&retmode=json&term=neoplasms[MeSH Terms] AND "epidemiologic methods"[MeSH Terms] AND (*Mesh Descriptor of Pesticide*[MeSH Terms] OR "Chemical Name of Pesticide"[Title/Abstract])
PubChem BioAssays (Active)	PubChem PUG	https://pubchem.ncbi.nlm.nih.gov/rest/pug/compound/cid/*PubChem Compound IDs*/aids/JSON?aids_type=active

Table S3. Cancer epidemiology papers retrieved from directed literature searches for parathion.

1: Alavanja MC, Hofmann JN, Lynch CF, Hines CJ, Barry KH, Barker J, Buckman DW, Thomas K, Sandler DP, Hoppin JA, Koutros S, Andreotti G, Lubin JH, Blair A, Beane Freeman LE. Non-hodgkin lymphoma risk and insecticide, fungicide and fumigant use in the agricultural health study. PLoS One. 2014 Oct 22;9(10):e109332. doi: 10.1371/journal.pone.0109332. eCollection 2014. PubMed PMID: 25337994; PubMed Central PMCID: PMC4206281.

2: Karami S, Andreotti G, Koutros S, Barry KH, Moore LE, Han S, Hoppin JA, Sandler DP, Lubin JH, Burdette LA, Yuenger J, Yeager M, Freeman LE, Blair A, Alavanja MC. Pesticide exposure and inherited variants in vitamin d pathway genes in relation to prostate cancer. Cancer Epidemiol Biomarkers Prev. 2013 Sep;22(9):1557-66. doi: 10.1158/1055-9965.EPI-12-1454. Epub 2013 Jul 5. PubMed PMID: 23833127; PubMed Central PMCID: PMC3773544.

3: Koutros S, Beane Freeman LE, Lubin JH, Heltshe SL, Andreotti G, Barry KH, DellaValle CT, Hoppin JA, Sandler DP, Lynch CF, Blair A, Alavanja MC. Risk of total and aggressive prostate cancer and pesticide use in the Agricultural Health Study. Am J Epidemiol. 2013 Jan 1;177(1):59-74. doi: 10.1093/aje/kws225. Epub 2012 Nov 21. PubMed PMID: 23171882; PubMed Central PMCID: PMC3590039.

4: Band PR, Abanto Z, Bert J, Lang B, Fang R, Gallagher RP, Le ND. Prostate cancer risk and exposure to pesticides in British Columbia farmers. Prostate. 2011 Feb 1;71(2):168-83. doi: 10.1002/pros.21232. Epub 2010 Aug 26. PubMed PMID: 20799287.

5: Dennis LK, Lynch CF, Sandler DP, Alavanja MC. Pesticide use and cutaneous melanoma in pesticide applicators in the agricultural heath study. Environ Health Perspect. 2010 Jun;118(6):812-7. doi: 10.1289/ehp.0901518. Epub 2010 Feb 17. PubMed PMID: 20164001; PubMed Central PMCID: PMC2898858.

6: Lee WJ, Sandler DP, Blair A, Samanic C, Cross AJ, Alavanja MC. Pesticide use and colorectal cancer risk in the Agricultural Health Study. Int J Cancer. 2007 Jul 15;121(2):339-46. PubMed PMID: 17390374; PubMed Central PMCID: PMC2928992.

7: Engel LS, Hill DA, Hoppin JA, Lubin JH, Lynch CF, Pierce J, Samanic C, Sandler DP, Blair A, Alavanja MC. Pesticide use and breast cancer risk among farmers' wives in the agricultural health study. Am J Epidemiol. 2005 Jan 15;161(2):121-35. PubMed PMID: 15632262.

8: Garcia SJ, Abu-Qare AW, Meeker-O'Connell WA, Borton AJ, Abou-Donia MB. Methyl parathion: a review of health effects. J Toxicol Environ Health B Crit Rev. 2003 Mar-Apr;6(2):185-210. Review. PubMed PMID: 12554434.

9: Waddell BL, Zahm SH, Baris D, Weisenburger DD, Holmes F, Burmeister LF, Cantor KP, Blair A. Agricultural use of organophosphate pesticides and the risk of non-Hodgkin's lymphoma among male farmers (United States). Cancer Causes Control. 2001 Aug;12(6):509-17. PubMed PMID: 11519759.

10: Pesatori AC, Sontag JM, Lubin JH, Consonni D, Blair A. Cohort mortality and

DOI: 10.1289/EHP186

nested case-control study of lung cancer among structural pest control workers in Florida (United States). Cancer Causes Control. 1994 Jul;5(4):310-8. PubMed PMID: 8080942.

11: Steensberg J. Health effects of chemical products. Ecol Dis. 1982;1(4):201-12. Review. PubMed PMID: 6236969.

12: Barthel E. [High incidence of lung cancer in persons with chronic professional exposure to pesticides in agriculture (author's transl)]. Z Erkr Atmungsorgane. 1976 Sep;146(3):266-74. German. PubMed PMID: 1023529.
